# Analyzing the Impact of Mobile App Engagement on Mental Health Outcomes: Secondary Analysis of the Unwinding Anxiety Program

**DOI:** 10.2196/33696

**Published:** 2022-08-15

**Authors:** William Nardi, Alexandra Roy, Shira Dunsiger, Judson Brewer

**Affiliations:** 1 Department of Behavioral and Social Sciences Brown University Providence, RI United States

**Keywords:** anxiety, worry, engagement, mobile app, mental health, mobile phone

## Abstract

**Background:**

App-based interventions provide a promising avenue for mitigating the burden on mental health services by complimenting therapist-led treatments for anxiety. However, it remains unclear how specific systems’ use of app features may be associated with changes in mental health outcomes (eg, anxiety and worry).

**Objective:**

This study was a secondary analysis of engagement data from a stage 1 randomized controlled trial testing the impact of the Unwinding Anxiety mobile app among adults with generalized anxiety disorder. The aims of this study were 2-fold: to investigate whether higher microengagement with the primary intervention feature (ie, educational modules) is associated with positive changes in mental health outcomes at 2 months (ie, anxiety, worry, interoceptive awareness, and emotional reactivity) and to investigate whether the use of adjunctive app features is also associated with changes in mental health outcomes.

**Methods:**

We analyzed the intervention group during the stage 1 trial of the Unwinding Anxiety mobile app. The total use of specific mobile app features and the use specific to each feature were calculated. We used multivariate linear models with a priori significance of *α*=.05 to investigate the impact of cumulative app use on anxiety, worry, interoceptive awareness, and emotional regulation at 2 months, controlling for baseline scores, age, and education level in all models. Significant relationships between system use metrics and baseline participant characteristics were assessed for differences in use groupings using between-group testing (ie, 2-tailed *t* tests for continuous data and chi-square analyses for categorical data).

**Results:**

The sample was primarily female (25/27, 93%), and the average age was 42.9 (SD 15.6) years. Educational module completion, the central intervention component, averaged 20.2 (SD 11.4) modules out of 32 for the total sample. Multivariate models revealed that completing >75% of the program was associated with an average 22.6-point increase in interoceptive awareness (*b*=22.6; SE 8.32; *P*=.01; 95% CI 5.3-39.8) and an 11.6-point decrease in worry (*b*=−11.6; SE 4.12; *P*=.01; 95% CI −20.2 to −3.1). In addition, a single log unit change in the total number of meditations was associated with a 0.62-point reduction in the Generalized Anxiety Disorder-7 scale scores (*b*=0.62; SE 0.27; *P*=.005; 95% CI −1.2 to −0.6), whereas a single log unit use of the stress meter was associated with an average of a 0.5-point increase in emotional regulation scores (Five Facet Mindfulness Questionnaire; *b*=0.5; SE 0.21; *P*=.03; 95% CI 0.1-0.9).

**Conclusions:**

This study offers a clearer understanding of the impact of engagement with app features on broader engagement with the health outcomes of interest. This study highlights the importance of comprehensive investigations of engagement during the development of evidence-based mobile apps.

## Introduction

### Background

As anxiety disorders (ADs) increase worldwide [1-5], app-based interventions offer evidence-based, high-fidelity treatment options [6] with incredible potential and comparable effects with traditional treatment models in efficacy trials [7]. Several recent meta-analyses that assessed app-based interventions for anxiety have indicated a growing evidence base [7-9]. A recent 2021 Nature review (*k*=22) [10] found significant but small effect sizes on anxiety symptomatology (Hedges *g*=0.2888; *P*<.001). These interventions can fill an important treatment gap, mitigate the growing burden on providers [11,12], and reduce barriers to care (eg, time, cost, and stigma) [13]. However, app-based interventions for AD often have low levels of user engagement, which reduces their efficacy and inhibits broader health care implementation [14]. To capitalize on the benefits of these interventions, engagement needs to be effectively understood and analyzed [10]. Specifically, research identifying the optimal levels of engagement with app-based interventions and the effects of various app features on anxiety-related outcomes is critical to realizing their full potential as treatments for AD [15-17].

To date, research on engagement remains limited and is often not assessed, as app-based interventions are still largely analyzed using conventional methods (eg, intent to treat) [18]. This method evaluates the effect of *assignment to treatment* (ie, the effect of being randomized to a group) instead of the direct effect associated with the app features that were actually used during the intervention [18]. Analyzing which features the user engages with is critical to designing personalized, effective interventions [14] and allows researchers to understand which in-app features drive changes in health outcomes [14,19]. Understanding engagement is also important for app development; specifically, it contributes fundamental information toward identifying the optimal intervention dose [17], streamlining inefficiencies [20], and tailoring apps to specific clinical populations [21]. Recognizing the importance of information on engagement, research is increasingly prioritizing the investigation of the relationship between engagement and improvements in mental health outcomes [10,16,22,23]. Encouragingly, in one of the first meta-analyses examining the effect of engagement on health outcomes, Gan et al [24] (*k*=25) found significant moderate improvements in postintervention symptomatology for participants categorized as having higher engagement than participants with lower levels of engagement (Hedges *g*=0.40; SE 0.16; 95% CI 0.097-0.705; *P*=.01). Although only 5 studies were designed to target AD, the effects indicated a significant positive association between engagement and anxiety (*r*=0.33; 95% CI 0.24-0.41; *P*<.001) [24]. This work is promising; however, the field remains preliminary and requires additional research to enhance the growing understanding of the associations between engagement with app-based interventions and anxiety [25-27].

Inconsistency in the definition and measurement of engagement is an ongoing challenge associated with analyzing the construct within this promising preliminary research [24]. Engagement can be broadly conceptualized as “(1) the extent (eg, frequency, duration) of usage; and (2) a subjective experience characterized by affect, attention, and interest” [28], as it relates to subsequent changes in the targeted health behavior (eg, anxiety and depression) [29,30]. However, different industries (eg, psychology and marketing) have historically focused on specific parts of this definition (eg, in-app use vs levels of engagement in the targeted health behavior), often failing to fully capture an understanding of the complex relationships that make up engagement [29,30]. To improve our understanding of app-based interventions and health outcomes, it is critical to understand the relationship between what is used during the intervention and subsequent changes in health outcomes [31]. One model proposed by Cole-Lewis et al [31] addresses this relationship and posits that engagement is multifaceted, encompassing multiple definitions from various disciplines. The model links both system-level and behavior-level engagement [31]. They defined engagement as a multidimensional construct encompassing a user’s interactions with app features that influence specific behavioral determinants, resulting in increased engagement in the targeted health behavior [31]. The model posits that the use of in-app features (ie, microlevel engagement) is directly associated with changes in the desired health outcome (ie, macrolevel engagement). Understanding both microlevel and macrolevel measurements is necessary to evaluate the effects of app-based interventions on improvements in mental health symptomatology [31].

Applying this model of engagement, we conducted a secondary analysis using multivariate regression models to examine the associations between in-app engagement (ie, microengagement) and mental health outcomes (ie, macroengagement) for participants with generalized AD (GAD) using a targeted mobile app called Unwinding Anxiety (UA) [32]. Data were collected from the intervention group in a recently published randomized controlled trial (RCT) that tested UA versus treatment as usual [32]. The UA app is a theory-driven, multifaceted app that comprises both guided (ie, educational modules) and unguided features (eg, meditations and ecological check-ins) targeting novel reinforcement learning constructs [32,33]. The results from the primary RCT (N=65) were promising, with participants in the UA group reporting a median reduction in anxiety scores of 8.5 (IQR 6.5; *P*<.001) and the treatment as usual group reporting a median reduction of 1 (IQR 5; *P*=.01), representing a 67% versus 14% reduction at the 2-month follow-up [32].

### Objectives

The aims of this study were 2-fold: (1) to investigate whether higher microengagement with the primary intervention feature (ie, educational modules) is associated with positive changes in mental health outcomes at 2 months (ie, anxiety, worry, interoceptive awareness, and emotional reactivity) and (2) to investigate whether the use of adjunctive app features is also associated with changes in mental health outcomes. We hypothesized that higher levels of microengagement with educational modules would be associated with significant changes in outcomes, which is consistent with improved mental health.

## Methods

### Overview

This study is a secondary data analysis of a previously described stage 1 parallel-group RCT [32]. As this secondary analysis is interested in system use data and their association with study outcomes, data were taken from the intervention arm only, and study procedures relevant to analyzing the intervention group are summarized in the following sections [32]. Roy et al [32] provide detailed information on the study design, procedures, and the results of the randomized trial.

### Ethics Approval

The primary trial was registered at Clinicaltrials.gov (NCT0368472), and the Brown University Institutional Review Board approved the study procedures (reference number PV4802) [32].

### Study Procedures

The data used for these analyses were from the baseline and 2-month time points for the intervention group only [32]. The inclusion criteria were as follows: (1) score ≥10 on the Generalized Anxiety Disorder 7-item scale (GAD-7), (2) owning a smartphone, (3) willingness to receive check-in calls, and (4) aged ≥18 years [32]. Participants were excluded from the study if they reported (1) dose changes of any psychoactive medication in the previous 2 months; (2) needed use of benzodiazepines and hypnotic sleep aids; (3) a history or current diagnosis of bipolar, schizophrenia or schizoaffective, or another psychotic disorder; (4) a significant medical condition that would affect the ability to complete study tasks; (5) cohabitation with someone already enrolled in the study; and (6) having a previous history of using other related apps, specifically Eat Right Now or Craving to Quit, which use similar reinforcement processes to UA [33-35]. Participants were recruited using social media largely through Facebook advertisements.

Eligible participants underwent informed consent procedures before enrolling in the study [32]. After enrollment, participants completed an in-person interview using the Mini International Neuropsychiatric Interview (MINI) International Neuropsychiatric Interview to confirm a diagnosis of GAD along with the assessment of other potential comorbid disorders (eg, depression, obsessive-compulsive disorder, and posttraumatic stress disorder) [32]. The participants were then asked to complete a web-based questionnaire using Qualtrics. Follow-up questionnaires were administered 2 months from treatment initiation using personalized email links specific to each participant’s unique identification number [32].

### Intervention

UA is an app-based intervention comprising educational modules that are considered the primary intervention features, consistent with recommendations from recent meta-analyses. The modules comprised instructional psychoeducational videos (5-15 minutes per day) teaching reinforcement learning concepts (Table 1). Modules are locked until the previous module is completed; however, participants can return to any of the already completed modules for review.

**Figure 1:**
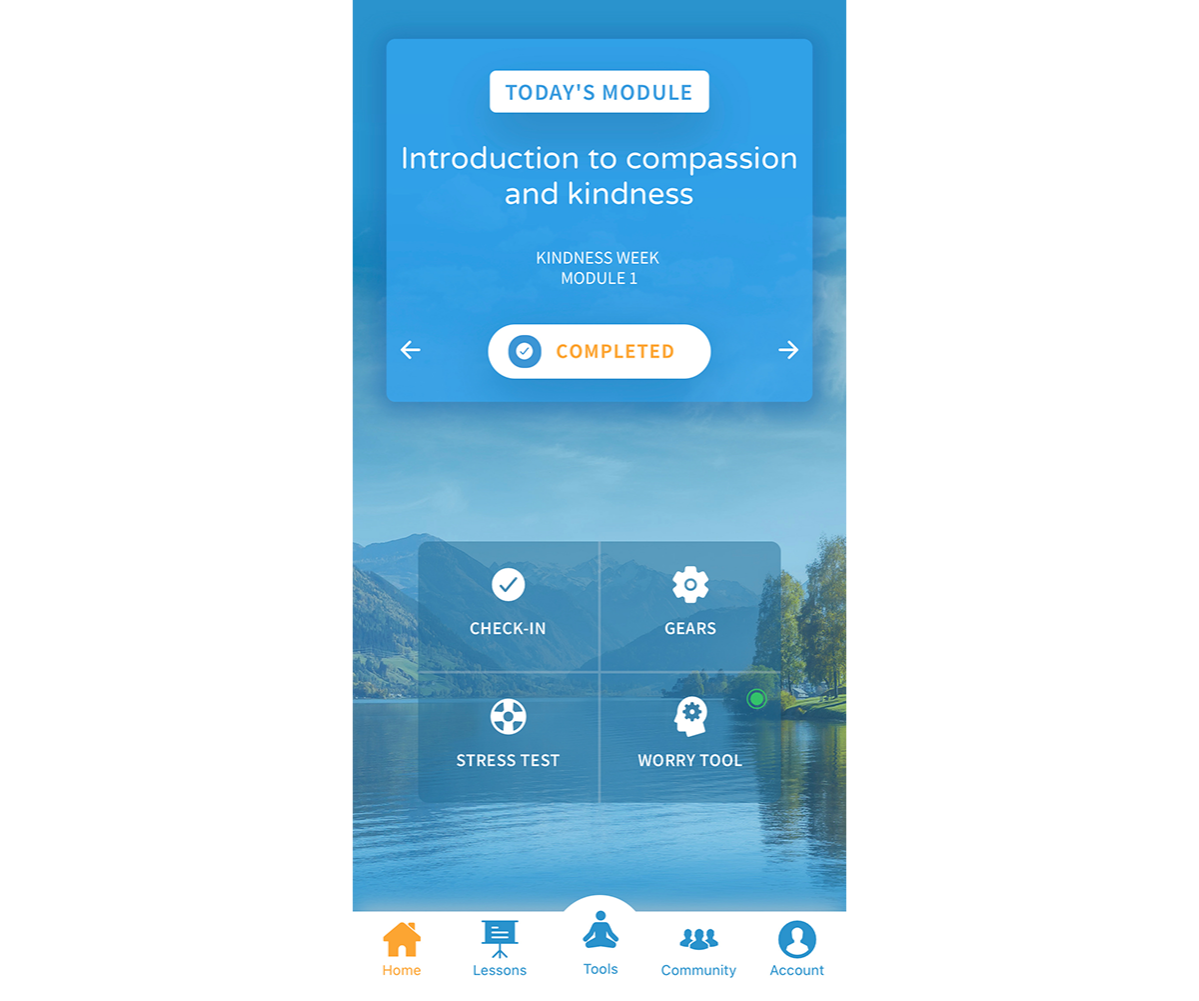
Unwinding Anxiety main dashboard.

**Figure 2:**
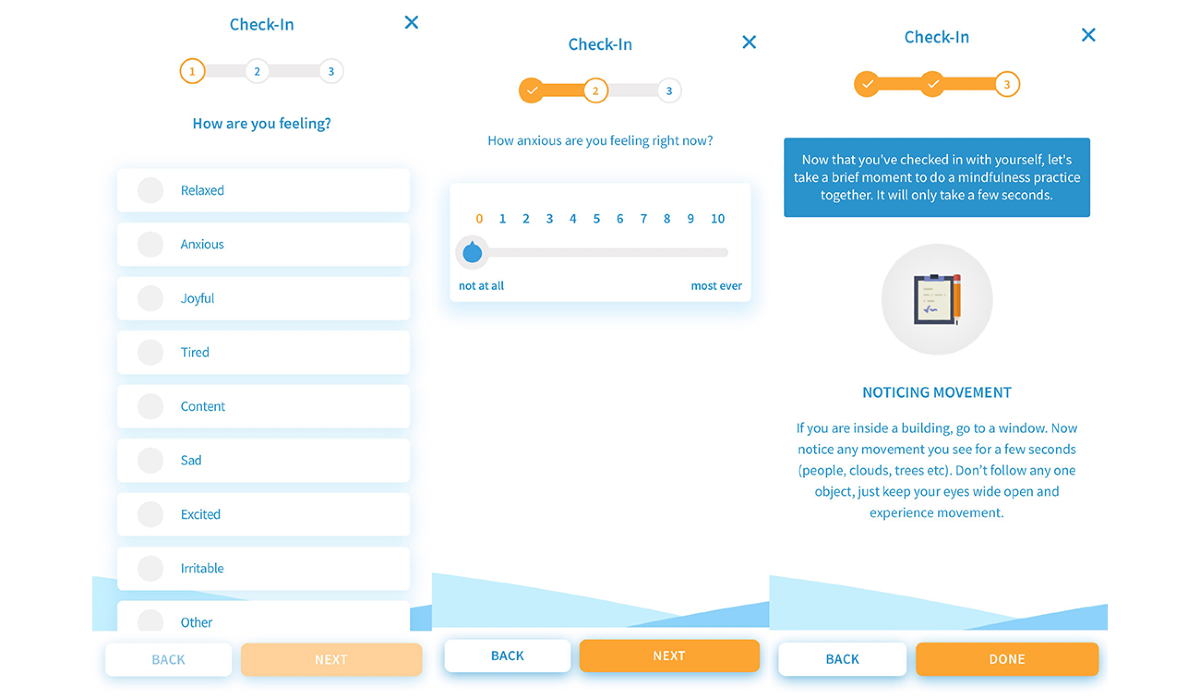
Adjunctive ecological feature example: check-ins.

In addition, the app offers unguided adjunctive features divided into 2 categories: ecological features designed to synergize with skills learned in the modules and meditation practices. The ecological features included physiological check-ins and 2 types of stress evaluation: a meter that evaluates the strength and reason for stress or anxiety and a stress test that assists in familiarizing participants with practicing curiosity regarding stress or anxiety using interoceptive awareness skills learned in the program. Detailed descriptions of these features can be found in Table 1, a visual depiction of the main dashboard is shown in Figure 1 and an example of an adjunctive ecological feature is provided in Figure 2. The example is the psychological check-in feature, in which a participant is asked first to identify their current emotional state from an initial list and then rate their anxiety level at the moment, and it ends by offering a recommendation for a short practice to return to present moment awareness depicted from left to right.

**Figure 3:**
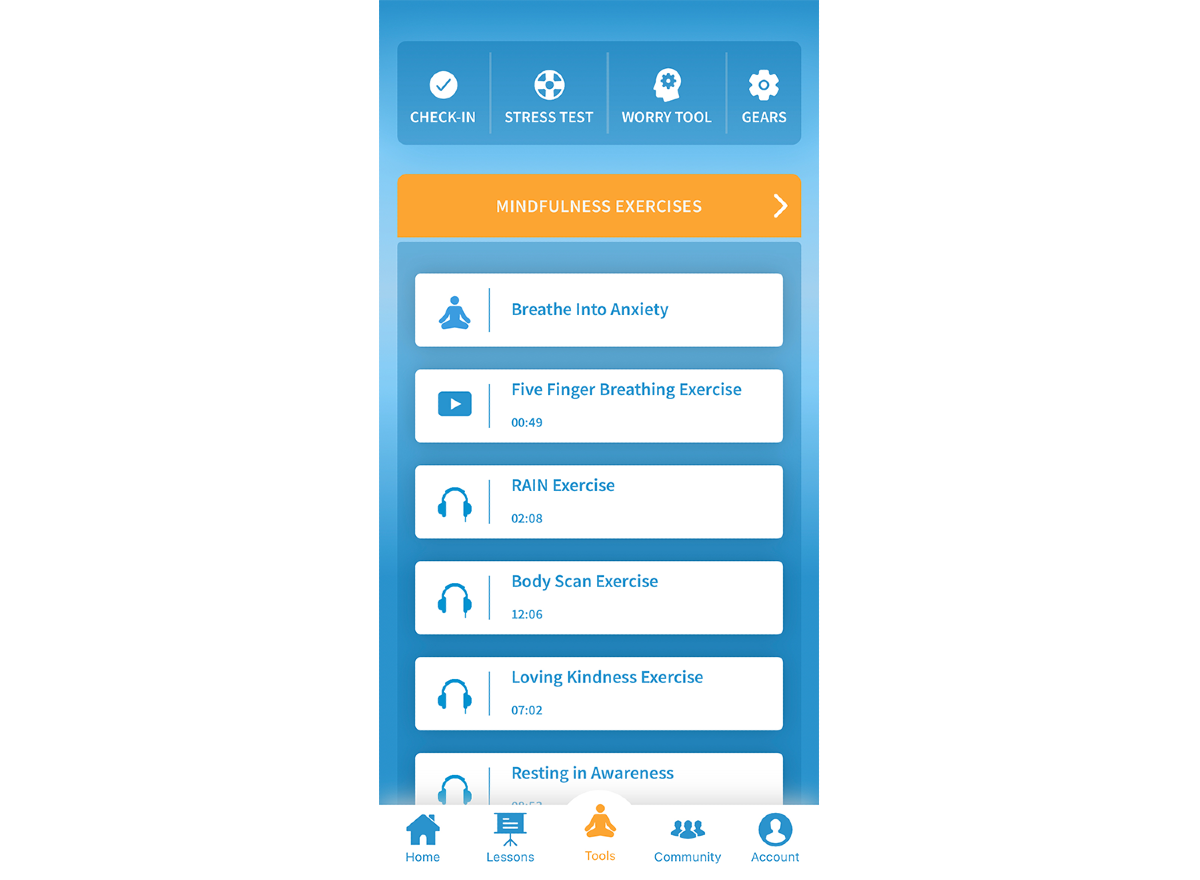
Adjunctive features dashboard.

**Figure 4:**
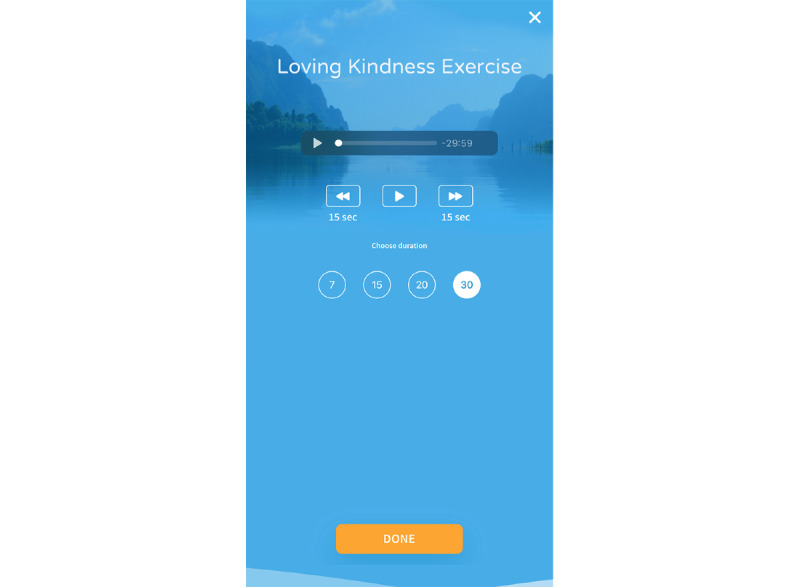
Adjunctive feature example: Loving Kindness meditation.

For the adjunctive meditation features, participants had access to a series of 3 practices that they were encouraged but not mandated to use: Resting in Awareness, a body scan practice, and the Loving Kindness practice (Table 1) [36]. Each meditation had the option to choose from 4 lengths of time (7-30 minutes). The intent of offering varying lengths of time is that participants initially engage in shorter practices and progress to longer periods of sustained meditation. A dashboard with meditation features is shown in Figure 3. An example of meditation (ie, Loving Kindness) is depicted in Figure 4.

**Table 1:** Overview of the Unwinding Anxiety app engagement features with content.

Feature or day introduced			Description
**Educational modules**			
	Modules 1-7, week 1	Overview of the program, personalized goal setting for the program that is logged in the app, and an introduction to the modules Topic areas focus on how worry and anxiety become habituated through reinforcement learning processes (ie, operant conditioning and reward-based learning), an overview of mindfulness and its application in identifying reinforcement patterns that result in negative health outcomes, and an introduction to curiosity as an attitudinal quality	
	Module 8-14, week 2	Introduction to the application of reinforcement concepts (ie, trigger, behavior, and reward), specifically, learning how to recognize behaviors, identifying the “rewards” or outcomes of the behaviors (eg, cognitive, physical sensations, and emotions), and becoming disenchanted with these behaviors allowing for an alternative behavioral pattern to emerge Novel to the program is the instruction to participants to not attempt to change behaviors immediately but to concentrate on the embodied experience (ie, interoceptive awareness and present moment awareness) of anxiety and the associated behaviors The modules introduce the RAIN^a^ practice and the role of curiosity in engaging with present moment experiences rather than judgment	
	Module 15-21, week 3	Week 3 begins with troubleshooting and applying reinforcement lessons from the previous week Participants are encouraged to gain acceptance of present moment experiences through resistance or unresistance, defined as the ability to engage with present moment experiences with curiosity, being aware of thoughts but not attached to them, and riding out waves of anxiety using the RAIN practice and other mindfulness exercises Modules then focus on the detriments of “contracting” or identifying with thoughts (ie, anxiety) Participants are asked to identify a variety of thought patterns (ie, anxiety, doubt, anger, and kindness) and observe rather than attach or react to these narratives	
	Module 22-30, week 4	The week begins with explaining the science of resistance to habit change, specifically, participants are introduced to the association between anxiety and performance (ie, anxiety becomes associated with accomplishing tasks) The previous modules regarding the application of mindfulness to unwind these associations are highlighted. Participants are guided through the importance of taking breaks when pursuing habit change, the advantages of alternate strategies to anxiety (ie, curiosity), and the ability to drop into the flow (ie, concentration and awareness focused on the present moment with a loss of reflective consciousness) The week ends with the key elements of continued motivation and review of the effectiveness of the program, specifically improvements participants have observed, termed “evidence-based faith”	
**Ecological features**			
	Check-ins, day 1	Select their current emotional state from a list provided (eg, happy, anxious, and relaxed) Describe the strength of their anxiety on a 10-point Likert scale (1=low and 10=high) Provided an exercise to complete (eg, hand awareness and breathe into anxiety)	
	Stress meter, day 1	Identify the strength of their anxiety on a 10-point Likert scale (1=low and 10=high) Identify from a list the reason for their anxiety (eg, uncompleted tasks and reliving past experiences) Provided with a short exercise to complete (eg, breathe into anxiety)	
	Stress test, day 6	Identify where anxiety is strongest in the body (eg, head, neck, and shoulders) Select a description of the sensation from a provided list (eg, tightness, pressure, and burning) Select the intensity from 0 to 100 on a scale (eg, 100=most stress ever) Identify on which side of the body the anxiety is strongest (ie, left or right) Provided with a short exercise to complete (eg, breathe into anxiety)	
**Meditations**			
	Resting in Awareness, day 1	Participants offer gratitude to themselves for taking the time to take care of themselves Subsequently, they are encouraged to shift their awareness to sounds in the room, then to thought processes by allowing thoughts to rise and pass away (ie, making a mental note of “thinking”) They are guided to directly observe thoughts as they arise and pass away on their own if not engaged with (eg, resisted) Subsequently, they are instructed to open their eyes, engaging in awareness of sights, sounds, thoughts, and body sensations, whichever present moment experience is most prominent in their experience Participants can choose from 9-, 15-, 20-, and 30-minute exercises	
	Body scan, day 3	Participants bring their attention to physical sensations in their body (eg, touch and pressure) and how sensations are connected to feelings or emotions (eg, anxiety) and are guided on how to pay attention to thoughts and mental processes (eg, noticing how “busy” thoughts can get when connected with anxiety) Participants can choose from 12-, 15-, 20-, and 30-minute exercises	
	Loving Kindness, day 5	Participants notice the physical sensations associated with an imagined experience of anxiety then shift to an experience when they meet a dear friend or kind being (ie, person and animal) From there, they are encouraged to investigate the different physical sensations of anxiety (eg, tightness and contraction) versus being with a kind person (eg, openness and warmth) They are then asked to offer phrases of kindness to the person or being identified (eg, “May you be happy”) using the phrases as mental anchors for present moment awareness Participants can choose from 7-, 15-, 20-, and 30-minute exercises	

^a^RAIN: Recognize and Relax, Allow and Accept, Investigate, and Note.

### Measures

#### Demographics

Demographic variables collected at baseline included age, biological sex, education level, and current employment status.

#### Psychiatric Diagnoses

The MINI is a short (15-minute) structured diagnostic interview assessing 17 of the most prevalent mental health disorders, including depression and ADs [37]. Previous studies have validated the MINI against the Diagnostic and Statistical Manual of Mental Disorders, Fifth Edition, and the International Classification of Diseases, Tenth Revision, and found the measure to be reliable and valid along with the added benefit of being more efficient [38].

#### Engagement Measures

The study measures were organized into 2 categories: microengagement and macroengagement. For clarity, in the model proposed by Cole-Lewis et al [31], engagement with the app-based intervention is referred to as the “little e” construct, which we categorize in these analyses as microengagement. The model then defines engagement with the targeted health outcome as the “Big E” construct, which we refer to as macroengagement [31].

#### Macroengagement: Mental Health Outcomes

##### Anxiety

Anxiety was assessed using the GAD-7, which comprises 7 items (eg, “feeling nervous, anxious, or on edge”). The GAD-7 is the most widely used self-report tool for clinical screening and tracking of GAD (sensitivity and specificity of 89% and 82%, respectively) [39]. Scores of ≥10 indicate a probable diagnosis of moderate GAD, whereas scores ≥15 indicate severe GAD [40]. In clinical testing, the scale demonstrated high reliability (Cronbach *α*=.88) and validity (*r*=0.69), correlated with the Beck Anxiety Index [41].

##### Emotional Regulation

The Five Facet Mindfulness Questionnaire (FFMQ) nonreactivity subscale was used to measure emotional regulation. The FFMQ is a validated 39-item questionnaire used to assess mindful awareness with high internal consistency (Cronbach *α* ≥.70) and an acceptable fit with a correlated 5-factor model (confirmatory fit index 0.914) [42]. It comprises 5 subscales, each validated for use independently [42,43]. The nonreactivity subscale comprises 7 questions (eg, “I perceive my feelings and emotions without having to react to them”) from the 39-item FFMQ with acceptable internal consistency (Cronbach *α*=.75) [32].

##### Interoceptive Awareness

The Multidimensional Assessment of Interoceptive Awareness is a 32-item measure with response options provided on a 6-point Likert scale (0=never and 5=always) that has demonstrated moderate levels of internal consistency (Cronbach *α* ≥.70) and a good model fit (comparative fit index 0.886) [44]. The scale comprises eight subscales: (1) noticing, (2) not distracting, (3) not worrying, (4) attention regulation, (5) emotional awareness, (6) self-regulation, (7) body listening, and (8) trusting (eg, “I trust my body sensations”) [44,45].

##### Worry

The Penn State Worry Questionnaire is a validated 16-item questionnaire used to assess worry (eg, “My worries overwhelm me”). It has high internal consistency (Cronbach *α*=.93) and validity compared with the Self-Analysis Questionnaire Tension subscale (*r*=0.36) and the Emotional Control Questionnaire (*r*=−0.53) [46]. Items are rated on a 5-point Likert scale (ie, 1=not at all typical and 5=very typical of me), and scoring for the scale is calculated as a total with possible ranges of 16 to 80 (eg, 60-80 indicating high worry) [46].

#### Microengagement: App Features Used

Microengagement (ie, system use) was defined as the total number of times each app feature was accessed [47]. The app features were organized into 3 categories for this study. The first category was engagement with the primary intervention feature and completion of the educational module. The other two engagement categories involved the use of the adjunctive app features: (1) ecological features and (2) meditations. These adjunctive or supportive features are components that participants could elect to use in combination with educational modules. Ecological features were considered adjunctive or supportive as they were designed as a short (<2 minutes in length) complement to the modules to assess participants’ current emotional or cognitive state at the moment. Meditations were defined as features >5 minutes in length, which followed evidence-based guidelines consistent with researched meditation practices (eg, Loving Kindness meditation) [48]. Table 1 provides a detailed description of each app feature. Aggregated totals for each of the 3 categories, as well as the total use of individual features, were calculated.

### Analysis

This was a secondary analysis of a previously conducted RCT. As such, the analysis was restricted to 32 participants who were randomized to the intervention group. A complete case analysis was conducted in this study. Of the 32 participants in the original intervention sample, 2 (6%) participants dropped out of the study before follow-up, and 2 (6%) participants did not complete the follow-up assessments, resulting in a final sample of 28 (88%) participants for this secondary analysis representing 88% of the original sample. For the primary RCT, a sample size of 52 was determined to have 80% power (1-sided Cronbach *α*=.05) to detect a statistically significant between-group difference in the primary outcome of anxiety (Cohen *d*=0.7). The final sample size was increased to 65 participants to account for 25% attrition.

Descriptive statistics, including means (SDs) for continuous variables and frequencies and percentages for categorical variables, were calculated for baseline variables, including demographics, anxiety, emotional regulation, and worry. Use data included ecological assessments (ie, check-ins, stress tests, and stress meters), mindfulness practices with the length of time completed (ie, Loving Kindness, Resting in Awareness, and body scan), and educational modules completed. Correlation analysis was used to identify potential confounders. Specifically, variables (eg, age, education, and income) were assessed using Spearman correlations, and those that met an a priori threshold of *P*≤.20 were included in subsequent models.

Microengagement was reported as the total number of times each app feature was accessed (ie, the total amount of use per item). Spearman rank correlation matrices were run to identify potential predictors to be included in the univariate linear regression models; predictors with a significance of *P*≤.20 were included [49]. Skewed engagement data were log transformed, except for the number of educational modules completed. Instead, the educational modules were dichotomized based on the completion of ≥75% (23/32) of the modules.

A series of multivariate linear regression models were constructed to investigate the dose-response relationships regarding the effect of use variables on psychosocial outcome measures. First, to determine predictors for inclusion in multivariate models, we ran a series of univariate linear models controlling for baseline scores to determine the impact of use variables on anxiety (ie, GAD-7), worry (ie, Penn State Worry Questionnaire), emotional regulation (ie, FFMQ), and interoceptive awareness (ie, Multidimensional Assessment of Interoceptive Awareness) at 2 months after treatment initiation. Predictors that were associated with outcomes at a modest *P*≤.20 level and met the assumptions testing criteria were then included in the subsequent multivariate linear regression models.

Final multivariate models investigated total meditation use, total ecological assessment use, individual meditation practices, and individual ecological assessments as predictors of the 4 outcomes (ie, anxiety, worry, interceptive awareness, and emotional regulation). All models controlled for baseline scores, as well as age and education level, as both have been shown to affect mobile app engagement in health behavior change in previous research [16]. Biological sex was not included as a covariate in the models to preserve parsimony because of a lack of correlation with outcomes (ie, *P*≥.20) in exploratory analyses and as only 7% (2/27) of the total sample identified as male versus 93% (25/27) as female. Analyses were run in R (version 4.0.3), and an a priori *α* level of .05 was set for the analyses. All models were evaluated to meet relevant model assumption criteria (eg, homoscedasticity and normality of residuals), and only models meeting the assumption criteria were reported. Summaries include *R*^2^ statistics (measure of effect size), *β* coefficients, 2-tailed *t* test values, *P* values, and 95% CIs for all the included models.

## Results

### Demographics

The participant demographics are presented in Table 2. The sample was primarily female (25/27, 93%), and the average age was 42.9 (SD 15.6) years. Most participants reported completing some level of college education and self-reported as employed: 67% (18/27) reported having attained a bachelor’s degree or higher, 59% (16/27) reported having full-time employment, and 74% (20/27) reported an annual income of ≥US $40,000 per year.

We explored potential differences in demographic information across high versus low engagement with the main intervention feature—education modules. High versus low engagement was quantified as those who completed >75% of the education modules (≥23 modules; high completion) and those who completed <75% (<23 modules; low completion). Both the high and low engagement groups averaged consistent numbers of participants who reported psychiatric conditions (Table 2). With one exception, the high engagement group had significantly more past depressive episodes (*P=*.02).

**Table 2:** Participant demographics: high versus low engagement and total sample (N=27).

Demographics		Low: <23 modules (n=13)	High: ≥23 modules (n=14)	*P* value	Total sample
Age (years), mean (SD)		37.7 (13.5)	47.7 (16.3)	.10	42.9 (15.6)
**Sex, n (%)**					
	Female	13 (100)	12 (86)	.50	25 (93)
	Male	0 (0)	2 (14)	.50	2 (7)
**Education, n (%)**					
	Some college or technical school	3 (23)	4 (29)	.90	7 (26)
	2-year degree	1 (78)	1 (7)	.90	2 (7)
	4-year degree	2 (15)	3 (21)	.90	5 (19)
	Master’s degree	7 (54)	5 (36)	.90	12 (44)
	Doctorate degree	0 (0)	1 (7)	.90	1 (4)
**Current employment, n (%)**					
	Employed full-time (≥35 hours weekly)	8 (62)	8 (57)	.10	16 (59)
	Employed part-time	3 (23)	0 (0)	.10	3 (11)
	Not in the labor force	1 (8)	5 (36)	.10	6 (22)
	Unemployed >1	1 (8)	1 (7)	.10	2 (7)
**Income (US $), n (%)**					
	20,000-29,000	1 (8)	1 (7)	.70	2 (7)
	30,000-39,000	2 (15)	1 (7)	.70	3 (11)
	40,000-49,000	2 (15)	1 (7)	.70	3 (11)
	50,000-59,000	1 (8)	0 (0)	.70	1 (4)
	70,000-79,000	1 (8)	1 (7)	.70	2 (7)
	80,000-89,000	1 (8)	1 (7)	.70	2 (7)
	100,000-149,000	4 (31)	4 (29)	.70	8 (30)
	>150,000	0 (0)	4 (29)	.70	4 (15)
**Mini International Neuropsychiatric Interview** **diagnostic criteria, n (%)**					
	Major depressive episode, current	3 (23)	0 (0)	.10	3 (11)
	Major depressive episode, past	2 (15)	9 (65)	.02^a^	11 (41)
	Panic disorder, current	2 (15)	1 (7)	.99	3 (11)
	Panic disorder, past	2 (15)	2 (14)	.99	4 (15)
	Posttraumatic stress disorder, met criteria	1 (8)	1 (7)	.99	2 (7)
	Obsessive-compulsive disorder, met criteria	2 (15)	2 (14)	.99	4 (15)
	Social anxiety disorder, met criteria	2 (15)	2 (14)	.99	4 (15)
	Agoraphobia, met criteria	1 (8)	4 (29)	.30	5 (19)

^a^Significance set at a priori *α* level of .05 (ie, *P*≤.05).

The baseline anxiety, emotional regulation, worry, and interoceptive awareness scores are reported in Table 3. Overall, the sample had average anxiety scores of 13.0 (SD 4.9) and high average worry scores of 65.5 (SD 7.1). The average interoceptive awareness score was 78.7 (SD 22.4), and participants had an average low emotional regulation score of 15.2 (SD 4.2). No significant differences were observed between the high and low engagement groups in the primary outcomes at baseline.

Participants completed an average of 20.2 (SD 11.4) modules of the primary intervention feature for the total sample. Of these, 52% (14/27) were considered “high completers” (ie, ≥23 modules). Use data of the adjunctive features over the 2-month intervention period are presented separately for high versus low engagement in Table 4. For the ecological features, participants’ total engagement averaged 38.1 (SD 52.6) uses. Regarding specific ecological features, the average check-in use accounted for most of the use at 31.1 (SD 45), whereas stress tests and stress meters averaged <5 uses for the 2-month period. When assessing adjunctive ecological feature engagement across high versus low primary intervention engagement, the high engagement group used adjunctive ecological tools more across all types of features than the low engagement group (Table 4).

Participants averaged 7.2 (SD 7.2) mindfulness practice uses over the 2-month period. The range of uses varied for meditations, although not as broadly as ecological tool use, with some individuals never using the meditations and other participants using the meditations more frequently, with median meditation use of 7 (IQR 8). In addition, when assessing meditation use across intervention engagement categories (ie, high vs low), those who completed a high level of the primary intervention also used meditation practices more on average (Table 4). Specifically, those who had a higher engagement with the primary intervention also used significantly more meditations overall, as well as the Loving Kindness meditation and Resting in Awareness meditation (Table 4).

Summary statistics of meditation practice use for the full sample, and for high versus low engagement, are presented in Table 3. The completion of cumulative mindfulness practices across the entire sample averaged 7.2 (SD 7.2) over the 2-month period. The range of uses varied for meditations, although not as broadly as the ecological tool uses, with some individuals never using the meditations and other participants using the meditations more frequently, with median meditation use of 7 (IQR 8). In reviewing differences in average meditation use between high and low engagement, the group with high engagement also used meditation practices more on average, and significant differences were observed for cumulative meditations used, Loving Kindness, and Resting in Awareness but not for the body scan meditations (Table 4).

**Table 3:** Average outcome measure scores at baseline.

Measure	Low: <23 modules, mean (SD)	High: ≥23 modules, mean (SD)	*P* value	Total sample, mean (SD)
Anxiety (Generalized Anxiety Disorder-7 scale)	13.4 (5.9)	12.6 (3.9)	.60	13.0 (4.9)
Emotional regulation (Five Facet Mindfulness Questionnaire, Nonreactivity Subscale only)	14.9 (4.2)	15.4 (4.4)	.70	15.2 (4.2)
Worry (Penn State Worry Questionnaire)	66.8 (6.6)	64.2 (7.6)	.30	65.5 (7.1)
Interoceptive awareness (Multidimensional Assessment of Interoceptive Awareness)	76.5 (19.6)	80.7 (25.2)	.60	78.7 (22.4)

**Table 4:** Average number of tool uses by segment over 2 months: high versus low engagement and total sample.

App components		Low: <23 modules, mean (SD)	High, ≥23 modules, mean (SD)	*P* value	Total sample, mean (SD)
**Cumulative ecological tool use**		12.9 (14.4)	61.6 (64.1)	.02^a^	38.1 (52.6)
	Check-ins	10.5 (12.0)	50.4 (55.5)	.02	31.1 (45.0)
	Stress test	1.2 (1.9)	4.9 (6.2)	.04	3.1 (4.9)
	Stress meter	0.5 (0.7)	2.2 (2.2)	.01	1.4 (1.9)
	Breath awareness	0.9 (1.9)	4.1 (5.4)	.05	2.5 (4.3)
**Cumulative mindfulness practice use**		3.1 (2.9)	10.9 (7.9)	<.01	7.2 (7.2)
	Loving Kindness Practice	1.0 (1.1)	4.2 (2.6)	<.01	2.7 (2.6)
	Body scan	1.1 (1.1)	3.4 (4.5)	.09	2.3 (3.5)
	Resting in Awareness	1.0 (1.4)	3.4 (2.8)	.01	2.2 (2.5)

^a^Significance set at a priori *α* level of .05 (ie, *P*≤.05).

### Multivariate Regression Results

#### Overview

Analyses were conducted to identify associations between microengagement, quantified as specific features use tools, and macroengagement, defined as worry, anxiety, interoceptive awareness, and emotional regulation. Predictors (ie, microengagement metrics) were entered into univariate models, controlling for baseline scores on the outcome of interest, and assumption testing was performed on models that met the a priori significance threshold (*P*≤.20). However, after review, models that violated assumptions were excluded, and those that met the criteria were consolidated for subsequent multivariate testing.

Using the results from the univariate models, multivariate linear regression models were built to answer the primary research question testing the association of (1) total meditation practice, (2) total ecological assessment, (3) individual meditation practices, and (4) individual ecological assessments with changes in psychosocial outcomes at 2 months (ie, anxiety, worry, emotional regulation, and interoceptive awareness). Only models that met a priori significance levels and all model assumptions are reported (Table 5) by system use categorization (ie, educational modules, ecological tools, and meditation practices). Parameter estimates, effect sizes (ie, coefficient of determination; *R*^2^), 95% CIs, and additional relevant statistics for all the included models are outlined in Table 5.

**Table 5:** Multivariate linear regression analyses of use metrics and change in psychosocial measures at 2 months.

Use metric^a^	Outcome	*R* ^2^	*P* value	*t* test (*df*)	*b* (95% CI)
Low vs high module	Interoception^b^	0.22	.01	2.72 (26)	22.6 (5.3 to 39.8)
Low vs high module	Worry^c^	0.25	.01	−2.83 (26)	−11.6 (−20.2 to −3.1)
Total meditations^d^	Anxiety^e^	0.21	.03	−3.16 (26)	−0.6 (−1.2 to −0.6)
Stress meter^d^	Mindfulness^f^	0.29	.03	2.36 (26)	0.5 (0.1 to 0.9)

^a^All models were adjusted for age, educational attainment, and baseline outcome measure total score.

^b^Multidimensional Assessment of Interoceptive Awareness.

^c^Penn State Worry Questionnaire.

^d^Variable was log transformed, and the results are reported on a log scale.

^e^Generalized Anxiety Disorder-7 scale.

^f^Five Facet Mindfulness Questionnaire, Nonreactivity Subscale only.

#### Education Modules

Completion of ≥75% of the educational modules was significantly associated with increases in interoceptive awareness and decreases in worry. More specifically, it was associated with an average 22.6-point increase in interoceptive awareness (SE 8.32; *P*=.01; 95% CI 5.3-39.8) and an 11.6-point decrease in worry (SE 4.12; *P*=.01; 95% CI −20.2 to −3.1) when holding age, education level, and baseline worry and interoceptive awareness scores constant.

#### Meditation Practices

Total meditation practice was associated with a significant reduction in anxiety scores. For each log unit change in the total number of meditations, there was a reduction of 0.62 in anxiety scores (SE 0.27; *P*=.005; 95% CI −1.2 to −0.6) after controlling for age, educational level, and baseline anxiety scores.

#### Ecological Tools

Stress meter use was associated with significant changes in emotional regulation. Specifically, a difference of 1 unit (on the log scale) in the stress meter was associated with a 0.5-unit increase on average in emotional regulation scores (SE 0.21; *P*=.03; 95% CI 0.1-0.9). Both associations were adjusted for age, education level, and baseline emotional regulation scores.

## Discussion

### Principal Findings

Comprehensive app-based interventions offer promising, high-fidelity treatments for AD, which are critical for addressing the growing treatment needs of the health care system [7,9,10,50]. To fully realize their potential, research will need to maximize engagement through a comprehensive understanding of how microengagement with in-app features affects macrolevel engagement in the target health outcome [31]. Our analyses sought to build on existing evidence by using multivariate linear regression models to examine the associations between improvements in mental health outcomes and the use of specific features within the UA mobile app. The results indicated that microengagement with app features was associated with significant changes in macroengagement in health outcomes (ie, interoceptive awareness, anxiety, emotional regulation, and worry), consistent with the engagement model proposed by Cole-Lewis et al [31]. Consistent with our hypothesis, engagement with the primary intervention feature (ie, completing at least 75% of the modules) was associated with an average increase of 22 points in interoceptive awareness scores and an 11-point decrease in clinical measures of worry at the 2-month follow-up. Associations were also found between the adjunctive app features (ie, ecological features and meditations) and improvements in health outcomes. Total meditation use was associated with a highly significant 0.62-point average reduction in anxiety (GAD-7), and the use of the Stress Meter, a tool for recognizing and investigating stressful situations in the moment, was associated with an average increase of 0.5 points in emotional regulation.

Moving forward, this research has significant implications for testing, developing, and customizing app-based interventions that target AD. First, this study offers important evidence that engagement with in-app features is a critical mechanism for increasing treatment benefits. Specifically, our findings showed a strong association between module completion and mental health outcomes, offering important evidence within the limited field of work on engagement with app-based interventions for AD. Previous research has largely focused on module completion as the primary engagement metric, finding small but significant associations with improvements in mental health outcomes for all digital interventions (eg, internet based and app based) [51-55]. However, these studies were mostly internet-based interventions, with few apps clinically tested for the effects of engagement on AD-related outcomes [23,24].

In addition, we found that intervention effects were driven by more than module completion, with findings indicating that adjunctive features (eg, ecological check-ins) were associated with important changes in health outcomes. The adjunctive or supportive components of complex app-based interventions have rarely been investigated. Instead, to date, most research has favored the analysis of completed modules or using a total frequency metric for all features used [24]. Focusing singularly on these adjunctive components offers 2 important insights: a clear indication of how those additional features are associated with changes in outcomes and information on potentially unnecessary or ineffective tools, which may needlessly complicate the app. Future research building on this study, focusing on understanding how specific supportive features function mechanistically to affect clinical outcomes, will be important as this information allows developers to streamline digital interventions [31]. By prioritizing streamlining app-based interventions, highlighting the effective features and removing ineffective features we can increase intervention efficiency as well as the likelihood of initiating and sustaining health behavior changes [22,56].

Although our analyses offer important novel insights into engagement and health outcomes, to fully capitalize on the potential of app-based interventions for sustained behavior change engagement, research needs to move beyond considering engagement as an aggregated total. Summed user engagement metrics, such as those used in these analyses, offer important insights but provide a limited view when considering the practical reality of engagement as a dynamic interaction between the participant and the app over time [22,23,47,57]. In the case of UA specifically, the app is designed to develop knowledge (eg, retraining reinforcement learning, education on understanding anxiety, and goal-directed behavior training) and promote skills maintenance (eg, meditation and emotional regulation), which can be applied in everyday life. The eventual goal of the app is actually “off ramping” participants or effectively reducing microengagement with the app while sustaining the associated health outcome improvements (eg, reductions in anxiety and increased emotional regulation). In this case, the pattern of engagement may change; for example, a participant may start at a higher level of engagement but begin to titrate how often they use the app over time. As such, the goal for UA is that health behavior change would be sustained despite lower engagement over time, which cross-sectional or sum engagement metrics cannot reflect. Thus, future analyses will need to move beyond aggregated engagement and analyze individual time series data to capture the dynamic, longitudinal relationship between in-app engagement and sustained health behavior changes.

### Comparison With Previous Work

Although the analysis presented is the first to investigate associations between the use of UA tools and mental health outcomes, the underlying reinforcement learning theory and curriculum for the primary intervention feature have been associated with behavioral reductions in our previous work on smoking cessation [58,59]. Specifically, our findings regarding module engagement build on results from an RCT (n=225) of the Craving to Quit program, a mobile platform designed to help people quit smoking, which uses a similar suite of educational modules grounded in the reinforcement learning theory and application of meditation practices on which the UA program is founded. These previous results indicated that educational module engagement, categorized as low (0-14 modules), medium (15-41 modules), and high (≥42 modules) in linear mixed models, was associated with significant reductions in the relationship between craving and cigarette smoking at 6 months (*F*_1,104_=4.44; *P*=.04) in the intervention group (n=182) [58].

In addition, in a previous trial of an app-based intervention targeting eating behaviors and craving-related eating—Eat Right Now—the results indicated significant associations between ecological feature engagement and craving-related eating [60]. Eat Right Now also comprises ecological features, which guide participants by investigating cravings, in addition to mindful eating exercises, to reduce the reward value of overeating [60,61]. The results showed significant effects of ecological feature use on reductions in craving-related eating, anticipated reward value of craving-related eating, and the likelihood of engaging in craving-related eating within an initial sample of 46 participants, which was then replicated among a larger sample (n=1119) using Rescorla-Wagner computational modeling [61].

This study, coupled with previous work, demonstrates the important role of multiple engagement metrics associated with positive changes in health outcomes among a range of clinical populations.

### Limitations

This study has some limitations. First, engagement data were calculated as the total number of uses. It is possible that a feature was opened, closed, or stopped midway. It is also possible that the participant was not actively engaged with the feature (eg, distracted or the participant was not the one using the app). In future studies, the length of use time should be considered for analysis to mitigate these limitations. In addition, this analysis considered specific features that operate singularly on health outcomes. However, this is unlikely to reflect the engagement patterns of participants who use a combination of features in pursuit of their health goals. Aggregation of microengagement patterns with the inclusion of the total time of engagement may offer windows into specific “usage profiles,” which could more effectively reflect the interdependent nature of many of these features. Another important limitation was the small sample size available for analysis. In addition, the sample was overwhelmingly female, White, and highly educated, making the generalizability of these findings to broader populations questionable. The length of follow-up may also have been a limiting factor for the analysis as there was variability regarding module completion, which may not have been aligned with the 2-month assessment window. Future analyses should use larger, more diverse samples to test the impact of microengagement patterns in the broader population.

### Conclusions

This secondary analysis offers evidence of associations linking the use of in-app features in UA to improvements in mental health outcomes; however, these results are preliminary and exploratory. The work presented offers a clearer understanding of the impact of how microengagement with the app features affects macroengagement with health outcomes of interest, consistent with the model proposed by Cole-Lewis et al [31]. This study highlights the importance of comprehensive investigations of engagement during the development of evidence-based app-based interventions. Future research would benefit from comprehensive, longitudinal analyses, as well as primary studies that specifically assess the effects of varying levels of engagement trials on health outcomes (eg, microrandomized trials, rapid optimization methods, and multiphase optimization strategies) [18,62-64]. As research continues, understanding the effects of engagement with app-based intervention features on clinical outcomes will be critical to designing targeted interventions needed to increase patient health and support accessible, comprehensive care.

## Abbreviations

AD: anxiety disorder
FFMQ: Five Facet Mindfulness Questionnaire
GAD: generalized anxiety disorder
GAD-7: Generalized Anxiety Disorder 7-item scale
MINI: Mini International Neuropsychiatric Interview
RCT: randomized controlled trial
UA: Unwinding Anxiety
